# Catheter Ablation in Arrhythmic Cardiac Diseases: Endocardial and Epicardial Ablation

**DOI:** 10.31083/j.rcm2309324

**Published:** 2022-09-19

**Authors:** Wen-Han Cheng, Fa-Po Chung, Yenn-Jiang Lin, Li-Wei Lo, Shih-Lin Chang, Yu-Feng Hu, Ta-Chuan Tuan, Tze-Fan Chao, Jo-Nan Liao, Chin-Yu Lin, Ting-Yung Chang, Ling Kuo, Cheng-I Wu, Chih-Min Liu, Shin-Huei Liu, Shih-Ann Chen

**Affiliations:** ^1^Heart Rhythm Center and Division of Cardiology, Department of Medicine, Taipei Veterans General Hospital, 11217 Taipei, Taiwan; ^2^Department of Medicine, School of Medicine, National Yang Ming Chiao Tung University, 112304 Taipei, Taiwan; ^3^Department of Medicine, Taipei Veterans General Hospital Taitung Branch, 95050 Taitung, Taiwan; ^4^Cardiovascular Center, Taichung Veterans General Hospital, 40705 Taichung, Taiwan

**Keywords:** arrhythmogenic left ventricular cardiomyopathy, arrhythmogenic right ventricular cardiomyopathy, Brugada syndrome, Chagas cardiomyopathy, left ventricular noncompaction, sarcoidosis

## Abstract

Arrhythmogenic cardiomyopathy (ACM) is a group of arrhythmogenic disorders of the myocardium that are not caused by ischemic, hypertensive, or valvular heart disease. The clinical manifestations of ACMs may overlap those of dilated cardiomyopathy, complicating the differential diagnosis. In several ACMs, ventricular tachycardia (VT) has been observed at an early stage, regardless of the severity of the disease. Therefore, preventing recurrences of VT can be a clinical challenge. There is a wide range of efficacy and side effects associated with the use of antiarrhythmic drugs (AADs) in the treatment of VT. In addition to AADs, patients with ACM and ventricular tachyarrhythmias may benefit from catheter ablation, especially if they are drug-refractory. The differences in pathogenesis between the various types of ACMs can lead to heterogeneous distributions of arrhythmogenic substrates, non-uniform ablation strategies, and distinct ablation outcomes. Ablation has been documented to be effective in eliminating ventricular tachyarrhythmias in arrhythmogenic right ventricular dysplasia (ARVC), sarcoidosis, Chagas cardiomyopathy, and Brugada syndrome (BrS). As an entity that is rare in nature, ablation for ventricular tachycardia in certain forms of ACM may only be reported through case reports, such as amyloidosis and left ventricular noncompaction. Several types of ACMs, including ARVC, sarcoidosis, Chagas cardiomyopathy, BrS, and left ventricular noncompaction, may exhibit diseased substrates within or adjacent to the epicardium that may be accountable for ventricular arrhythmogenesis. As a result, combining endocardial and epicardial ablation is of clinical importance for successful ablation. The purpose of this article is to provide a comprehensive overview of the substrate characteristics, ablation strategies, and ablation outcomes of various types of ACMs using endocardial and epicardial approaches.

## 1. Introduction

Arrhythmogenic cardiomyopathy (ACM) has a variety of definitions and classifications. ACM is defined from a narrow perspective as a genetically mutated form of cardiac muscle disease that features fibrofatty changes of the right and/or left ventricles [1, 2]. From a broader perspective, such as that of the latest Heart Rhythm Society (HRS) expert consensus, ACM is a disease entity characterized by diseased myocardium that is not caused by ischemic, hypertensive, or valvular heart disease [3, 4]. As a result, ACM encompasses a wide range of diseases, including arrhythmogenic right ventricular cardiomyopathy (ARVC), arrhythmogenic left ventricular cardiomyopathy (ALVC), cardiac amyloidosis, cardiac sarcoidosis, Chagas cardiomyopathy, Brugada syndrome (BrS), and left ventricular noncompaction (LVNC) [4]. Additionally, several ACMs were progressive in nature. Consequently, the manifestation of ACM in late stages can overlap with that of idiopathic dilated cardiomyopathy, further complicating the identification of underlying etiologies [4]. Antiarrhythmic drugs (AADs) are commonly used to treat ventricular tachyarrhythmia, including ventricular tachycardia/fibrillation (VT/VF) in patients with ACM. Nevertheless, AADs were frequently constrained by their inefficacy and well-documented toxicities. In recent years, as our understanding of the underlying pathogenesis and ablation technologies improved, radiofrequency catheter ablation (RFCA) has been implemented as an alternative therapy for VT/VF in ACM [1].

Several studies have demonstrated that ventricular tachyarrhythmia can be eliminated by ablation in patients with ARVC, sarcoidosis, Chagas cardiomyopathy, and Brugada syndrome (BrS) [5, 6, 7, 8, 9]. The heterogeneity of substrate characteristics can also result in different ablation strategies and outcomes for ACMs. Since VT circuits in ACM are commonly distributed three-dimensionally, both endocardial and epicardial approaches are frequently required to achieve a successful ablation [10]. The present article reviews the latest evidence regarding the endocardial and epicardial ablation for various types of ACMs and the associated ablation outcomes.

## 2. Arrhythmogenic Substrates in ACM

### 2.1 Substrate Characteristics in Various Types of ACM

Contrary to ischemic cardiomyopathy, in which arrhythmogenic substrates are usually confined to the endocardium [5], there is often a discrepancy in arrhythmogenic substrates between the epicardium and the endocardium in ACM, regardless of the underlying cause [6, 7, 8, 9].

#### 2.1.1 Arrhythmogenic Right Ventricular Cardiomyopathy

ARVC is by far the most comprehensively documented ACM. The first report was published in 1982, which led to the development of an international guideline for diagnosis and treatment [11]. The majority of ARVC has been identified as an inherited autosomal dominant disease characterized by an abnormality of cell-to-cell adhesion. Histopathologic findings are characterized by the progressive replacement of fibro-fatty tissue within the right ventricle (RV), ultimately resulting in VT with a left bundle branch block (LBBB) morphology [4, 12, 13].

ARVC was originally described as a primarily RV disease [11]. Recent improvements in imaging modalities, such as late gadolinium enhancement cardiac magnetic resonance (LGE-CMR), have demonstrated that fibro-fatty infiltration and replacement are not limited to the RV. Therefore, biventricular and left ventricular-dominant variants have been described [14]. While the RV-dominant variant, commonly abbreviated as “ARVC”, does not involve the left ventricle (LV), the LV-dominant variant, usually referred to as “ALVC”, does not reveal any RV abnormalities. In the biventricular variant, both RV and LV abnormalities can be observed [14]. Typically, the arrhythmogenic scar attributable to ARVC is located at the so-called “triangle of dysplasia”, which includes the tricuspid annulus (TA) and the RV outflow tract (RVOT) and could extend to the RV free wall and apex. The scar most commonly affects the epicardium first, then gradually involves the endocardium as the disease progresses [12, 14, 15, 16].

#### 2.1.2 Arrhythmogenic Left Ventricular Cardiomyopathy

The current consensus on the diagnosis of ALVC is based on the international Padua criteria, which encompasses major and minor criteria regarding structural/functional dysfunction, repolarization abnormalities, VT/VF, and genetics in the same manner as the diagnosis of ARVC [17]. In contrast to the mutant genetics encoding desmosomal proteins in ARVC, the mutant genetics in ALVC are primarily involved in non-desmosomal genes such as lamin A/C, phospholamban, and filamin-C [1, 4, 18, 19, 20].

**Fig. 1.**
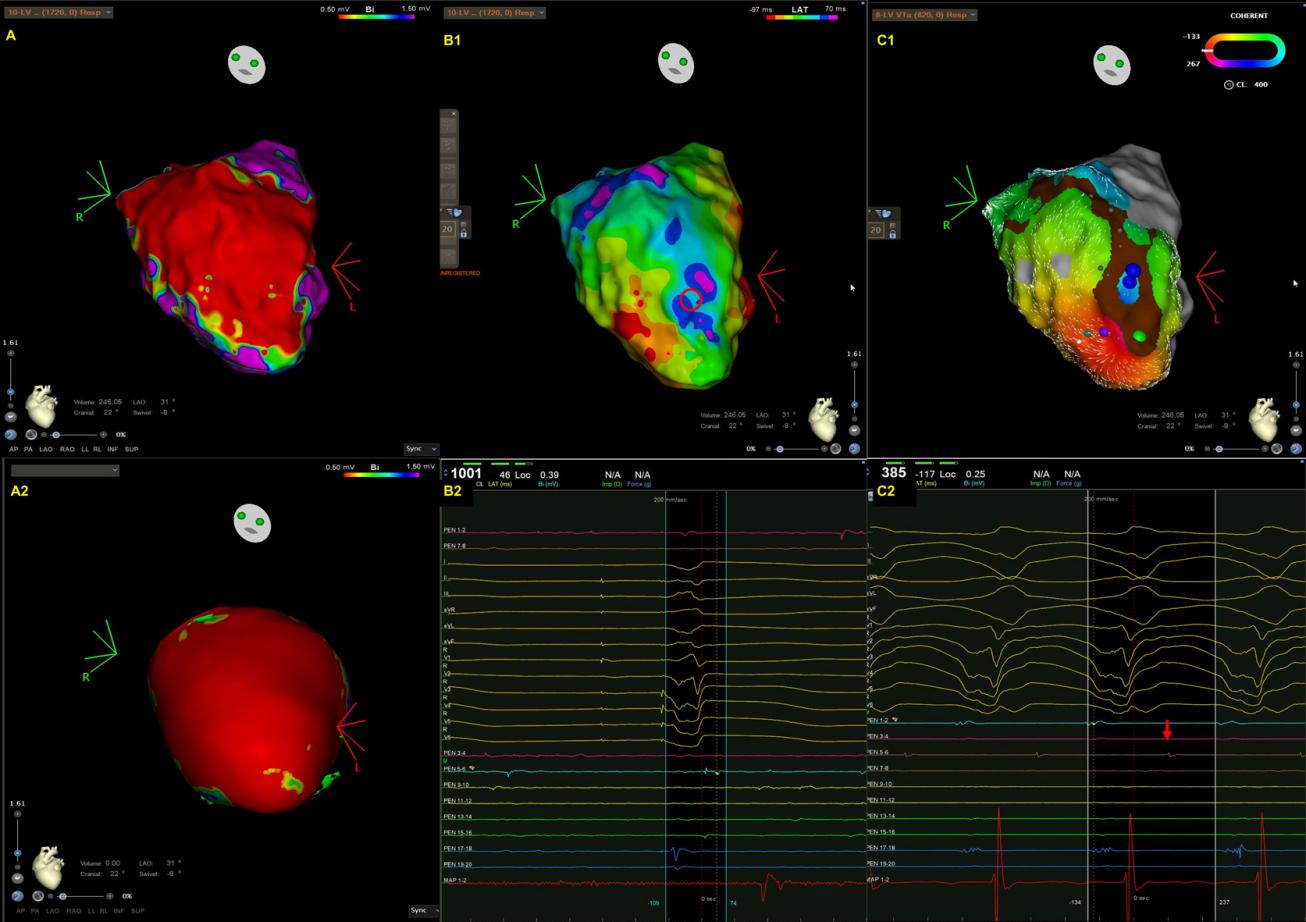
**A representative case of intramural ventricular tachycardia (VT) in a patient with arrhythmogenic left ventricular cardiomyopathy (ALVC) caused by titin mutation**. Endocardial bipolar voltage (A) and preprocedural cardiac MRI (A2) revealed extensive scarring along the anterior septum and anterior wall, extending from the base of the LV to its apex. The scars identified by cardiac MRI are larger than those detected by endocardial bipolar voltage mapping owing to the presence of intramural scarring. (B1–2) An isochronal late activation map (B1) created by annotating the latest component of bipolar electrogram during right ventricular pacing and fractionated potential (B2) recorded within scar (red circle in B1). In particular, an isochronal crowding region was noted close to the scar border in the LV apex. (C1–2) VT activation map (C1), VT morphology and diastolic potential (C2). VT cycle length was 385 milliseconds with a left bundle branch block morphology and superior axis. The VT activation map (from red, orange, yellow, green, blue, indigo, to violet) demonstrated an incomplete circuit characterized by an activation gap (parts of the blue and violet are missing) within the endocardium. The mid-diastolic potential (red arrow, C2) was recorded at the blue dot site (C1). After radiofrequency energy was applied to the blue dot area, VT was terminated.

As opposed to ARVC, where the arrhythmogenic substrate is typically located at the triangle of dysplasia in the RV [16], ALVC exhibits fibrofatty infiltration along the LV posterobasal and anterolateral walls [21] (Fig. 1). Since the LV wall is thicker than that of the RV, the arrhythmogenic substrate tends to stay in subepicardial layers without expanding to the subendocardium [1, 22].

It should be emphasized that ARVC and ALVC tend to be progressive in nature and this fact should be taken into account when considering RFCA.

#### 2.1.3 Cardiac Amyloidosis

Amyloidosis is an infiltrative disease that occurs as a result of abnormally folded proteins deposited on the myocardium [23]. There are two major subtypes of amyloidosis: light chain (AL) amyloidosis and transthyretin (ATTR) amyloidosis [23]. The clinical manifestations of cardiac involvement include diastolic dysfunction, disease of the small vessels, conduction system disease, and atrial and ventricular arrhythmias (VA) [4, 24, 25, 26, 27, 28]. Arrhythmogenic substrates in cardiac amyloidosis are typically located in the non-coronary artery territory of the LV, either transmural or subendocardial in nature [29]. In addition, there are different patterns of LGE in cardiac amyloidosis subtypes, such that ATTR amyloidosis has a more extensive transmural substrate and RV involvement than AL amyloidosis [30]. It is noteworthy that cardiac amyloidosis can also mimic ARVC in presentation, and an endomyocardial biopsy may be necessary for diagnosis [4, 31]. Variable scar distribution patterns can be observed as the disease progresses, including localized, patchy, and subepicardial LGE [32].

#### 2.1.4 Cardiac Sarcoidosis

Sarcoidosis is an inflammatory disease characterized by granulomatous infiltration throughout multiple organs [33]. Once the cardiovascular system is involved, the clinical manifestations can be variable, ranging from none to advanced heart failure and sudden cardiac death (SCD) [23]. In spite of the fact that only 5% of sarcoidosis patients manifest clinical symptoms of cardiac involvement, autopsy reveals that up to 25% of patients have cardiac sarcoidosis involvement [34].

**Fig. 2.**
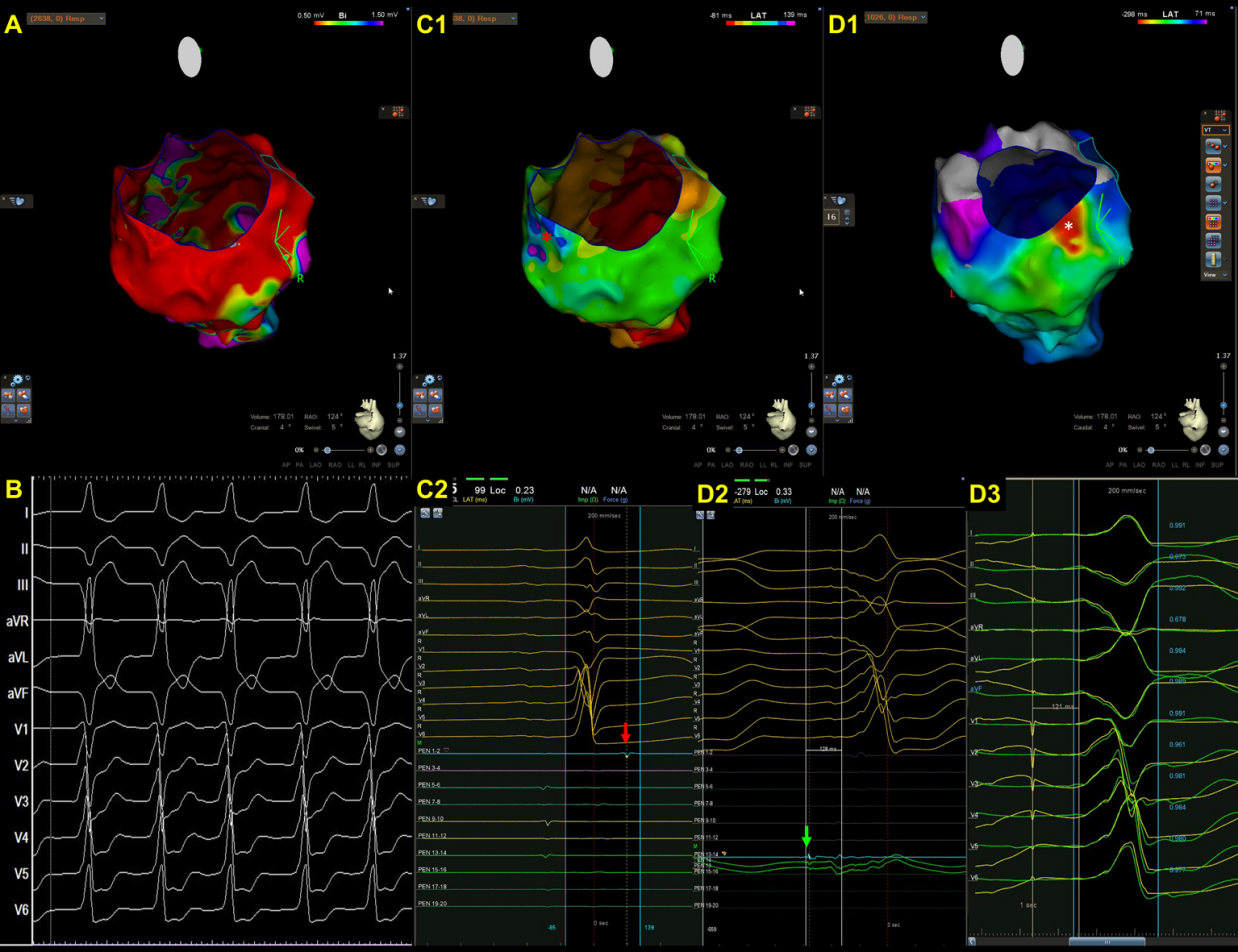
**A representative case of focal VT in a patient with cardiac sarcoidosis**. (A) An endocardial bipolar voltage map showed extensive scarring of the LV septum and inferior wall. (B) The spontaneous VT was characterized by a left bundle branch block pattern and superior axis. Wobbling of the VT cycle lengths was also noted. (C1–2) An isochronal late activation map (C1) was created by annotating the latest component of a bipolar electrogram. At the basal inferior portion of LV, there was an isochronal crowding region. (C2) The red asterisks indicate an isolated late potential (red arrow in C1) (D1–3). VT activation map (D1), the earliest electrogram during VT mapping (D2), and pacemapping at the earliest activation site (D3). The VT activation maps (D1, from red, orange, yellow, green, blue, indigo, to violet). Contrary to the majority of scar-related VTs, there was a centrifugal pattern of activation in this patient, indicating that the mechanism of VT in this case was focal rather than macroreentrant. There was a very early prepotential (D2, green arrow) preceding the onset of VT by 128 ms in the white asterisks area (D1) where was adjacent to the isochronal crowding region. During pacemapping, the QRS morphology at the earliest activation point was 96% similar to that of clinical VT. There was also a significant delay between the stimulus and QRS onset (121 ms) during pacemapping. The VT was terminated by ablation at this site.

Cardiac sarcoidosis can affect RV, LV, or both [35, 36, 37, 38]. Patchy scarring is most often observed in the septum, followed by the anterior wall, the LV outflow tract, the inferior wall, the lateral wall, and the apex within the mid-myocardial and subepicardial layers of the LV, whereas scarring is generally seen in the RV [35, 36]. In addition, since the basal septum is frequently involved, right septal VTs, peritricuspid/perimitral VTs, or VTs originating from the Purkinje system are also common [37, 39]. A representative case was shown in Fig. 2.

#### 2.1.5 Chagas Cardiomyopathy

Chagas disease is caused by the protozoan Trypanosoma cruzi, which is responsible for the highest disease burden of any parasite in the Western Hemisphere [40]. If left untreated and accompanied by cardiac involvement, Chagas disease can lead to dilated cardiomyopathy that results in heart failure, VAs, and conduction system dysfunction [40]. Chagas cardiomyopathy is defined as cardiac involvement with at least a typical electrocardiographic abnormality [40].

A Chagas cardiomyopathy is characterized by necrosis and fibrosis of the myocardium with disruptions of the intercellular junctions, which are usually located at the basal inferolateral walls of the LV and the ventricular aneurysms [41, 42, 43]. The extent of myocardial fibrosis and substrates depends on the stage of the disease. Notably, approximately one-third of VT circuits could be identified on the epicardium [44, 45].

#### 2.1.6 Brugada Syndrome

The majority of BrS is an inherited autosomal dominant disease [46], which is characterized by the presence of coved type J-point elevation in the right precordial leads on electrocardiography (ECG) [47]. SCD is often caused by VT/VF in BrS [48, 49]. As per current consensus, the diagnosis of BrS is made if an ECG shows an ST-segment elevation of greater than 2 mm in one or more of the right precordial leads, V1 and/or V2, positioned at the second, third, or fourth intercostal spaces, either spontaneously or in response to given sodium-channel blockers (e.g., ajmaline, flecainide, procainamide, or pilsicainide) [50]. The genes encoding sodium channel, calcium channel, and potassium channel are mostly associated with BrS [51]. Among all genetics discovered, reduced INa and loss-of-function *SCN5A* gene mutations are the most important ones, accounting for 20 to 30% of BrS [51, 52].

The arrhythmogenic substrate of BrS, which includes electrical and structural abnormalities, is predominantly located in the anterior epicardial region of RVOT [47, 49, 53, 54, 55]. It should be noted that since BrS could be dynamic and progressive, a gradient of collagen deposition can often be observed between the epicardium and endocardium. This suggests a progressive development of the arrhythmogenic substrate from the epicardium to the endocardium [53]. Interestingly, with the provocative drug test, the arrhythmogenic substrate increased as RV function worsened, particularly in the anterior free wall of RVOT [56].

#### 2.1.7 Left Ventricular Noncompaction

LVNC is a genetic disease that results in the developmental arrest and failure of the heart during the final phase of cardiac development, which is featured by excessive and unusual trabeculations in the LV [4, 57, 58]. The morphological anomaly is typically seen as a spongy appearance of the myocardium in the LV, with abnormal trabeculations mainly located in the apical, mid-lateral, and inferior portions of the LV [4]. Although it is often referred to as LVNC, RV involvement has also been documented, resulting in RV noncompaction or biventricular non-compaction [59, 60].

Since ventricular involvement is heterogeneous, noncompaction cardiomyopathy can be subclassified into nine subtypes, including the most benign form, the RV form, the biventricular form, the dilated cardiomyopathy form, the hypertrophic cardiomyopathy form, the restrictive cardiomyopathy form, a mixed form, the congenital heart disease form, and the arrhythmogenic form [4, 59, 61]. The diagnosis of LVNC is based on non-invasive image modalities such as echocardiography and LGE-CMR, which can reveal the maximal ratio of the thickness between the non-compacted layer and the compact layer thickness, evidence of intertrabecular recesses filled in the LV cavity by color Doppler echocardiography, and segmental localization of hypertrabeculation indicative of non-compaction [4]. However, it should be noted that hypertrabeculation itself is not necessarily a disease [4].

In LVNC, the arrhythmogenic substrate is frequently seen in the endocardium and epicardium of the left and right ventricles at the site of the outflow tracts, Purkinje system, and scarring similar to that of dilated cardiomyopathy [62].

### 2.2 When to Consider the Epicardial Approach?

As discussed above, since three-dimensional circuits are frequently observed in VTs and epicardial substrates are commonly observed in ACMs, successful ablation usually requires both endocardial and epicardial approaches [10]. As a result, the decision to perform the epicardial approach is crucial, and it is dependent upon both pre-operative and intraoperative information.

A non-invasive evaluation prior to RFCA might be helpful. ECGs are the most commonly used tool in ACM and are often used as the first step in locating ventricular tachyarrhythmias. Thus, it would be worthwhile to examine the relationship between ECG characteristics and arrhythmogenic substrates. Traditionally, ECG characteristics such as a pseudo-delta wave more than 34 ms [63], an intrisicoid deflection time more than 85 ms [63], an RS complex duration more than 121 ms [63], a maximum deflection index more than 55 [64], and presence of inferior q waves [65] are considered to be indicators of epicardial origin for VT.

Nevertheless, since the included patients were diverse and were not limited to patients with ACM, it is important to apply the aforementioned criteria with caution [63, 64, 65]. Furthermore, different ECG patterns suggest that epicardial origin may vary depending on the ACM entity. It is noteworthy that the correlation between ECG and epicardial substrates is widely studied in ARVC.

In patients with ARVC, Bazan *et al*. [66] reported that an inferior or anterior Q wave or QS pattern of VT was suggestive of the requirement of an epicardial approach. In addition, Kubala *et al*. [67] demonstrated that more advanced transmural substrates could be detected if downsloping elevated ST-segments were observed in V1 and V2, indicating the need for an epicardial approach. In previous studies, we demonstrated that inter-lead QRS dispersion of precordial leads was associated with the requirement of epicardial ablation [68]. Furthermore, the presence of J waves in the inferior leads was related to the discrepancy between endocardial and epicardial activation [69]. We also found the diagnostic criteria of ARVC based on signal-averaged ECG could also help predict the need for epicardial ablation [70].

In addition to ECGs, advanced imaging modalities such as cardiac computed tomography and LGE-CMR are important to assess the requirement of epicardial approaches [71]. Indeed, based on the distribution of LGE, the need for an epicardial approach could be made before RFCA [72]. In cardiac sarcoidosis, since granulomatous infiltration can involve any region of the myocardium, the utility of positron emission tomography (PET) is noteworthy and helpful in assessing the disease burden [36, 38, 73, 74, 75, 76].

Aside from the non-invasive evaluation described above, an intraoperative assessment may also suggest that an epicardial approach is necessary. In intracardiac echocardiography imaging, increased echogenicity could be detected in diseased regions and was associated with myocardial scarring [77]. Besides, it has been demonstrated that endocardial unipolar voltage mapping can be used to reliably identify epicardial arrhythmogenic substrates during substrate mapping [78, 79]. In addition, the absence of isochrones within the diastolic path during reentrant VT circuits in the endocardium also suggests intramural or epicardial circuits [10].

## 3. Mapping of Ventricular Tachyarrhythmias and Ablation Strategies

Despite the fact that ventricular tachyarrhythmias have several mechanisms, including automaticity, triggered activity, and macroreentry, macroreentry is usually the predominant mechanism in ACMs [4, 71]. Delineation of critical isthmuses of VT is crucial to VT elimination and provides more favorable results [80].

Ideally, the most important step is to induce clinically documented VT. We applied rapid ventricular pacing and programmed stimulation of up to three extra-stimuli from the RV apex and/or RVOT to induce VT in our laboratory [12, 81, 82]. When VTs are induced, QRS morphology and cycle lengths, either as documented by 12-lead ECG or intracardiac defibrillator (ICD), have been compared with those of clinically documented VTs [12, 81, 82]. Once the VTs are induced and mappable, activation mapping and entrainment mapping are employed to illustrate the VT circuits and identify critical isthmuses [83, 84]. It is noteworthy that since three-dimensional circuits are frequently observed in VTs and epicardial substrates are frequently observed in ACMs, an incomplete circuit characterized by an activation gap (Fig. 1) or endocardial/epicardial focal centrifugal activation pattern could be discovered [10]. Therefore, entrainment from the earliest activation sites and the adjacent scar is required to determine the potential exit or surrogate of reentrant circuits [85].

In cases where VTs are non-inducible or unmappable for reasons such as hemodynamic instability or changing morphologies/cycle lengths, alternative strategies, such as substrate modifications that eliminate local abnormal ventricular activity, isolated delayed component ablation, scar dechanneling, may also provide promising results [86, 87, 88]. Recently, functional substrate mapping seems to have become more relevant to VT critical isthmus [89]. Moreover, the application of multi-electrode catheter for VT mapping is based on isochronal late activation maps, which demonstrated favorable ablation results [90, 91, 92].

Considering that three-dimensional circuits and epicardial substrates are often present in ventricular tachyarrhythmias, it is noteworthy that simultaneous epicardial and endocardial recordings are frequently essential for RFCA of these tachyarrhythmias [10, 93].

## 4. Outcomes of RFCA in Different Entities of ACM

The current guidelines emphasize that RFCA is reserved for patients with a high burden of ventricular ectopy and non-sustained VT as well as recurrent sustained VT in symptomatic and drug-refractory ACM patients. This treatment is not considered a definitively curative treatment [4, 71]. Ablation of VT in ACM patients is intended to eliminate or reduce the arrhythmogenic substrates that are fundamental in the development of reentrant VT [4]. Since ACMs are heterogeneous, the outcome of RFCA is also determined by the disease process and evolving arrhythmogenic substrate [4].

### 4.1 RFCA Outcomes in ARVC and ALVC

The evidence on the effectiveness of RFCA in ARVC is extensive and well documented compared to other ACM. As a consequence of the limitations in understanding the disease and the technology, studies prior to 2009 have relatively few outcomes. These studies are limited to a small number of patients who have undergone endocardial-based ablation [94, 95, 96]. In these patients, 25–53% of patients were free from recurrence of VT after ablation [94, 95, 96].

As the disease is investigated more thoroughly and with the improvement of technologies, a discrepancy of arrhythmogenic substrates is often noticed between the epicardium and endocardium, resulting in the need for an epicardial approach [6]. The use of an endocardial and epicardial approach improved the freedom from VT recurrence to 47–95% in the following studies [6, 16, 69, 97, 98, 99, 100, 101, 102, 103, 104, 105, 106, 107]. As a result of the analysis of the ARVC Program at Johns Hopkins, which included 116 patients and 166 procedures, Daimee *et al*. [107] reported that RFCA could lead to VT-free survival with 68.6% and 49.8% at 1 and 5 years, respectively, after a single procedure and multiple procedures could further lead to VT-free survival with 81.8% and 69.6% at 1 and 5 years, respectively (Table 1, Ref. [6, 12, 16, 69, 94, 95, 96, 97, 98, 99, 100, 101, 102, 103, 104, 105, 106, 107]). On the contrary, since isolated cases of ALVC are relatively uncommon, limited data can be found to assess the outcome of RFCA.

**Table 1.** **Summary of Clinical Outcomes of VT ablation in ARVC patients**.

Clinical studies	Study aim	Mapping and/or ablation sites	Number of patients	Age	Acute success	Major complications	Follow-up	Short-term VA recurrences (≤1 year)	Long-term VA recurrences
Verma *et al*. (2005) [94]	To report the results and success of substrate-based VT ablation	Endocardial alone	22	41 ± 15 years	82%	1 patient with cardiac tamponade	Median of 37 months	23%	47%
Satomi *et al*. (2006) [95]	To examine the relationship between the reentrant circuits of VT and the abnormal electrograms in ARVC, and to assess the feasibility of a block line formation in the reentrant circuit isthmus utilizing electroanatomical mapping system guidance	Endocardial alone	17	47 ± 17 years	88%	No complications	26 ± 15 months	NA	23.5%
Dalal *et al*. (2007) [96]	To evaluate the outcomes of radiofrequency catheter ablation of VT in ARVC patients	Endocardial alone	24	36 ± 9 years	Total procedural success: 46%; Partial procedural success: 31%	1 patient with procedure-related death	32 ± 36 months	50%	75%
Garcia *et al*. (2009) [97]	To characterize the endocardial versus epicardial substrate, measure right ventricular free wall thickness, and determine epicardial ablation efficacy in patients with ARVC	Endocardial & Epicardial	13	43 ± 15 years	92%	No complications	18 ± 13 months	NA	23%
Bai *et al*. (2011) [98]	To compare the long-term freedom from recurrent VAs by using endocardial-alone ablation versus endo-epicardial substrate-based ablation	Group 1: Endocardial alone	49	Group 1: 34 ± 14 years; Group 2: 37 ± 11 years	All patients achieved the procedural end point at the end of ablation	No major complications	Group 1: 1224 ± 310 days; Group 2: 1175 ± 112 days	NA	Group 1: 47.8%; Group 2: 15.4%
		Group 2: Endocardial & Epicardial	Group 1, n = 23; Group 2, n = 26						
Philips *et al*. (2012) [99]	To assess the efficacy of radiofrequency catheter ablation of VT in ARVC, with particular focus on newer ablation strategies, including epicardial catheter ablation	Endocardial ± Epicardial	87	38 ± 13 years	Complete success 47%; Partial success 38%	1 patient with procedure-related death; 1 patient with delayed myocardial infarction	88.3 ± 66.1 months	53%	85%
Philips *et al*. (2015) [100]	To report procedural strategy, safety, and efficacy of epicardial radiofrequency catheter ablation with a focus on the characteristics of the substrate and recurrent VT	Endocardial ± Epicardial	30	33.1 ± 11.1 years	97%	No major or minor complications	19.7 ± 11.7 months	24%	30%
Santangeli *et al*. (2015) [101]	To determine the long-term outcomes of VT control and need for antiarrhythmic drug therapy after endocardial and adjuvant epicardial substrate modification in patients with ARVC	Endocardial ± Epicardial	62	39 ± 15 years	VT noninducibility was achieved in 77% patients	2 patients with DVT and pulmonary embolism; 1 patient with pericardial effusion; 1 patient with RV puncture; 1 patient with constrictive pericarditis	56 ± 44 months	NA	29%
Müssigbrodt *et al*. (2017) [102]	To examine the long-term results of an inducibility-guided ablation strategy in a large cohort of patients with ARVC	Endocardial ± Epicardial	70	53.2 ± 14.0 years	VT noninducibility was achieved in 84.4% patients	1 transient ischemic attack, 2 acute pericardial effusions; 2 pulmonary thromboembolisms (one lethal) later during the hospital stay	31.1 ± 27.4 months	NA	42.2%
Wei *et al*. (2017) [103]	To summarize radiofrequency catheter ablation for recurrent drug-refractory VTs due to ARVC	Endocardial ± Epicardial	48	39.9 ± 12.9 years	81.3%	No major complications	71.4 ± 45.7 months	NA	43.7%
Kirubakaran *et al*. (2017) [16]	To characterize the RV substrate using electroanatomical mapping and to define outcomes following VT ablation in patients with and without RV structural abnormalities	Endocardial ± Epicardial	29	Group 1: 38 ± 10 years; Group 2: 47 ± 16 years	VT noninducibility was achieved in 93% in Group 1 and 87% in Group 2	No major complications	22 ± 11 months	NA	27%
			Group 1: electrical cardiomyopathy (n = 14); Group 2: structural cardiomyopathy (n = 15)						
Lin *et al*. (2018) [104]	To investigate the prognostic value of scar distribution in patients with ARVC	Endocardial ± Epicardial	80	47 ± 15 years	100%	2 patients with pulmonary edema; 1 patient with pseudo-anuerysm	38 ± 11 months	5%	48.8%
Souissi *et al*. (2018) [105]	To investigate relevant radiofrequency ablation outcomes in a multicentric registry	Endocardial ± Epicardial	49	47 ± 13 years	71%	1 patient with cardiac tamponade, hemothorax and DVT; 1 patient with femoral arterio-venous fistula; 1 patient with intestinal perforation	64 ± 51 months	63%	86%
Mathew *et al*. (2019) [106]	To investigate the sequential approach for VT ablation in patients with ARVC	Endocardial ± Epicardial	47	44 ± 16 years	Complete success 80%; Partial success 16%	1 patient with cardiac tamponade	Median follow-up of 50.8 months	37%	55%
Santangeli *et al*. (2019) [6]	To determine the long-term outcomes of catheter ablation of VT in a series of patients with ARVC without background implantable cardioverter-defibrillator therapy	Endocardial ± Epicardial	32	45 ± 13 years	VT noninducibility was achieved in all patients	1 patient with RV laceration	Median follow-up of 46 months	NA	19%
Lin *et al*. (2021) [69]	To investigate the significance of J waves with respect to substrate manifestations and ablation outcomes in patients with ARVC	Endocardial ± Epicardial	45	Group 1: 51.8 ± 12.9 years; Group 2: 44.2 ± 13.7 years	Successful ablation was achieved in all patients	No major complications	33.9 ± 23.0 months	NA	15.6%
			Group 1: with J wave (n = 13); Group 2: without J wave (n = 32)						
Daimee *et al*. (2021) [107]	To provide new insights on clinical outcomes based on a large series of VT ablation procedures from the current era in ARVC patients	Endocardial ± Epicardial	116	Median of 34.3 years	Total procedural success: 95.8%; Partial procedural success: 4.2%	1 patient with delayed pericardial effusion	5.2 ± 3.2 years	Single procedure: 31.4%; Multiple procedure: 18.2%	Single procedure: 50.2%; Multiple procedure: 30.4%

ARVC, arrhythmogenic right ventricular cardiomyopathy; DVT, deep vein thrombosis; RV, right ventricle; NA, not applicable; VA, ventricular arrhythmia; VT, ventricular tachycardia. This table is modified from Cheng *et al*. [12].

### 4.2 RFCA Outcomes in Cardiac Amyloidosis 

In cardiac amyloidosis, atrial arrhythmias are more common than ventricular arrhythmias. No large-scale studies have been conducted to evaluate the ablation outcomes of VA in cardiac amyloidosis [108, 109].

Mlcochova *et al*. [110] reported that in two patients with repetitive electrical storms caused by focal monomorphic ventricular ectopy, RFCA could effectively prevent recurrences of the storms. No abnormal endocardial substrates were observed in this case report [110]. In our previous report, we described a 53-year-old man who had multiple episodes of VT. The patient was later diagnosed as multiple myeloma-related cardiac amyloidosis, which was finally confirmed by endomyocardial and bone marrow biopsy [111]. In this case, voltage mapping revealed extensive scarring on both the endocardium and epicardium from RVOT to the basal free wall of the RV. Abnormal electrograms within these areas were targeted and eliminated, and no recurrence of VAs was noted during follow-up at 6 months [111].

### 4.3 RFCA Outcomes in Cardiac Sarcoidosis 

In the previous six observational studies with limited case numbers, the degree and phase of cardiac sarcoidosis varied widely. Therefore, the long-term efficacy of RFCA cannot be generalized [36, 37, 38, 39, 75, 112]. The recurrence rate of VT is approximately 13%–75% [36, 37, 38, 39, 75, 112]. Muser *et al*. [38] analyzed the largest group of patients, consisting of 31 patients. Endocardial and epicardial mapping/ablations were performed in 8 patients, with a VA recurrence rate of 52% after a mean follow-up of 2.5 years [38]. A recent systematic review evaluated the effectiveness and outcomes of VT ablation based on the results of 5 clinical trials that involved 83 patients [113]. All patients received endocardial ablation, while 18% underwent epicardial ablation [113]. In almost all studies, VA freedom was achieved in nearly 55% of patients, and burden reduction in 88% (or more) of patients [113] (Table 2, Ref. [36, 37, 38, 39, 75, 112]).

**Table 2.** **Summary of Clinical Outcomes of VA ablation in CS patients**.

Clinical studies	Study aim	Mapping and/or ablation sites	Number of patients	Age	Acute success	Major complications	Follow-up	VA recurrences
Koplan *et al*. (2006) [112]	To define the electrophysiologic characteristics of the VT and its electrophysiologic substrate	Endocardial ± Epicardial	8	42 ± 8 years	82%	NR	6 months to 7 years	75%
Jefic *et al*. (2009) [39]	To assess the response of VT in patients with CS to medical therapy and radiofrequency ablation	Endocardial ± Epicardial	9	46.7 ± 8.6 years	70%	NR	19.8 ± 19.6 months	44%
Dechering *et al*. (2013) [75]	To investigate whether there are significant demographic and electrophysiological differences between patients with CS and ARVC	NR	8	mean age 44.9 years	63%	NR	6 months	13%
Naruse *et al*. (2014) [37]	To describe both clinical and electrophysiological characteristics and outcomes of systematic treatment approach to VT associated with CS	Endocardial alone	14	56 ± 11 years	79%	NR	33 months	43%
Kumar *et al*. (2015) [36]	To examine the ventricular substrate and outcomes of catheter ablation	Endocardial ± Epicardial	21	47 ± 9 years	90%	4.7%	4.8 ± 5.1 years	71%
Muser *et al*. (2016) [38]	To determine the long-term outcome of catheter ablation of VT in patients with CS	Endocardial ± Epicardial	31	55 ± 10 years	NR	4.5%	2.5 years	52%

ARVC, arrhythmogenic right ventricular cardiomyopathy; CS, cardiac sarcoidosis; NR, not reported; VA, ventricular arrhythmia; VT, ventricular tachycardia.

### 4.4 RFCA Outcomes in Chagas Cardiomyopathy

The effectiveness of RFCA has been evaluated in Chagas cardiomyopathy. After 35 months of follow-up, 92.1% of patients with electrical storm and 60.5% of patients with VT had been free from the electrical storm and VT in a prospective study with 38 patients (16 with Chagas cardiomyopathy) receiving RFCA [114].

As in other ACM, reentry is the main mechanism of VT in Chagas cardiomyopathy. In addition, an inferolateral scar is found in over 70% of patients with Chagas cardiomyopathy [71] and is often located in the intramyocardial layer along with a thick layer of subendocardial myocardium [115, 116], leading to approximately 37% prevalence of epicardial VT origins [71]. Although endocardial RFCA can sometimes successfully create transmural lesions and eliminate VT effectively, epicardial mapping and ablation are often required in up to 40% of patients [71].

According to previous studies, the combined endocardial and epicardial approach to Chagas cardiomyopathy demonstrated a significant decrease in the recurrence of VA without increased major complications in comparison to endocardial ablation alone [117, 118, 119, 120, 121]. Moreover, in a recent randomized controlled trial, Pisani *et al*. [122] enrolled 30 patients with Chagas cardiomyopathy undergoing VA ablation and divided them into two groups in a 1:1 ratio: one group underwent combined endo-epicardial ablation and the other underwent an endocardial ablation approach. The acute success, defined as the absence of inducible clinical VT, was achieved in 86% of patients in group 1 and only 40% in group 2. After a median follow-up of 587 days, VT recurrence occurred in 40% and 80% of patients in group 1 and group 2, respectively [122]. There were no differences in perioperative complications reported between these two groups [122].

### 4.5 RFCA Outcomes in Brugada Syndrome

The effectiveness of RFCA in BrS has not been evaluated by randomized controlled trials. On the basis of previous observational studies, 73–100% of patients were free from recurrent VA during follow-up [123, 124, 125, 126]. Fernandes *et al*. [127] performed a systematic review encompassing 11 case series and 11 case reports with a total of 233 patients and reported that the success rates of VA ablation over a 2.5–7.8 follow-up period were 96.7%, 70.6%, and 80% with epicardial, endocardial, and triggering ventricular ectopy ablation approaches, respectively. More than 77.3% of patients in these studies required an epicardial approach [127]. It is significant to note that in 92.9% of patients with combined epicardial and endocardial mapping, there was no identifiable endocardial substrate, therefore epicardial mapping and ablation were necessary [127]. The most commonly ablated area was the anterior epicardial RVOT, followed by the anterior RV, inferior RV, and lateral TA on the epicardium [127].

The provocation test with sodium channel blocker or epicardial warm water instillation was shown to enhance Brugada phenotype, epicardial arrhythmogenic substrates, and VA [127, 128]. Brugada *et al*. [125] first demonstrated the value of sodium channel blockers by demonstrating significantly increased epicardial arrhythmogenic substrates and VA inducibility after flecainide provocation. In a similar manner, Zhang *et al*. [124] utilized procainamide to enhance low-voltage zones, the elevation of the J-point and ST segment, and transmural dispersion of late activation. Pappone *et al*. [126] conducted an ajmaline provocation test to determine the degree of coved ST-elevation and epicardial arrhythmogenic substrates.

In our previous experience, we described a novel method for identifying functional epicardial substrates using epicardial warm water instillation [128]. In this cohort, we analyzed 15 type 1 BrS patients with VT who received RFCA [128]. Consistent with results from other studies, significantly larger epicardial arrhythmogenic substrates were found at RVOT and the anterior RV free wall [128]. In six patients, epicardial warm water instillation enlarged the arrhythmogenic substrates and increased VA inducibility [128].

In summary, RFCA (especially the combined epicardial and endocardial approach) seems to be safe, feasible, and provides favorable outcomes in BrS. A pharmacologic or a warm water provocation test can be considered to identify potential arrhythmogenic substrates (Table 3, Ref. [123, 124, 125, 126, 127, 128, 129, 130, 131, 132, 133, 134, 135, 136, 137, 138]).

**Table 3.** **Summary of Clinical Outcomes of VA ablation in BrS syndrome patients**.

Clinical studies	Study aim	Mapping and/or ablation sites	Number of patients	Age	Type I Brugada pattern elimination	Major complications	Follow-up	VA recurrences
Nademanee *et al*. (2011) [123]	To investigate whether the substrate site is the RVOT in patients with BrS who have frequent recurrent VF episodes	Endocardial ± Epicardial	9	39 ± 10 years	89%	2 patients with pericarditis	20 ± 6 months	11%
Shah *et al*. (2011) [129]	Case report	Endocardial alone	1	43 years	100%	0%	78 months	0%
Sunsaneewitayakul *et al*. (2012) [130]	To observe the feasibility of substrate modification by radiofrequency catheter ablation and its effects on VF storm	Endocardial alone	4	24 ± 3 years	100%	1 patient with RBBB	12–30 months	75%
Cortez-Dias *et al*. (2013) [131]	Case report	Endocardial ± Epicardial	1	60 years	100%	NR	6 months	0%
Szeplaki *et al*. (2014) [132]	Case report	Endocardial ± Epicardial	1	31 years	100%	NR	18 months	0%
Maeda *et al*. (2015) [133]	Case report	Endocardial ± Epicardial	1	38 years	NR	NR	20 months	0%
Forkmann *et al*. (2015) [134]	Case report	Endocardial ± Epicardial	1	22 years	NR	NR	9 months	0%
Brugada *et al*. (2015) [125]	To systematically report the methodology, results, and complications of epicardial ablation of consecutive selected patients with BrS	Endocardial ± Epicardial	14	37 ± 8 years	100%	1 patients with pericarditis	3–6 months	0%
Notarstefano *et al*. (2015) [135]	Case report	Endocardial alone	1	39 years	NR	NR	18 months	0%
Zhang *et al*. (2016) [124]	To investigate the mechanism and arrhythmogenic substrate of VT/VF and to evaluate the long-term outcomes of catheter ablation in patients with BrS	Endocardial ± Epicardial	11	48 ± 16 years	100%	2 patients with pericarditis	25 ± 11 months	27%
Saha *et al*. (2016) [136]	Case report	Endocardial ± Epicardial	1	34 years	NR	1 patients with pericarditis	41 months	0%
Tauber *et al*. (2016) [137]	Case report	Endocardial alone	1	38 years	100%	NR	NR	0%
Hayashi *et al*. (2016) [138]	Case report	Endocardial alone	1	37 years	0%	NR	6 months	100%
Chung *et al*. (2017) [128]	To elucidate the thermal effect on BrS phenotype, VT/VF, and electrophysiological characteristics of epicardial functional substrates in BrS	Endocardial ± Epicardial	15	41 ± 10 years	63.6%	NR	3–6 months	7%
Pappone *et al*. (2017) [126]	To investigate the methodology and results of substrate-based mapping/abla-tion in a large series of consecutive BrS patients with various clinical presentations and to verify if radiofrequency ablation could normalize the consequences of a genetic disease	Endocardial ± Epicardial	135	39–40 ± 10–12 years	98.5%	5 patients with pericarditis	10 months	1.5%

BrS, Brugada syndrome; NR, not reported; RBBB, right bundle branch block; RVOT, right ventricular outflow tract; VA, ventricular arrhythmia; VF, ventricular fibrillation; VT, ventricular tachycardia. This table is adopted and modified from Fernandes *et al*. [127].

### 4.6 RFCA Outcomes in LVNC

As LVNC is a rare and heterogeneous disease, a limited number of cases and inconsistent results could be expected. RFCA has been demonstrated in previous case reports [139, 140, 141, 142] as well as in small observational cohort studies [62, 143, 144, 145] to be a safe and feasible method of managing VT. For these studies, mapping and/or ablation of the epicardium and endocardium were often required for satisfactory outcomes [62, 139, 141, 143, 144, 145]. Muser *et al*. [143] reported in a study of 9 patients (1 patient with combined endocardial and epicardial mapping and ablation) that the arrhythmogenic substrates of VT were localized in the mid-apical segments of the LV and the origin of ventricular ectopies were from papillary muscles and/or basal septal regions. RFCA led to 89% freedom from VA recurrence after a median follow-up of four years. In a study of 18 patients (two of whom underwent combined endocardial and epicardial mapping and ablation), Li *et al*. [144] found that VT circuits in RVOT, TA, anterolateral papillary muscle, and inferolateral wall were located. The success rate of RFCA was 85.7% after the mean follow-up of 54 months. In a multi-center observational study consisting of 18 patients (2 patients with combined endocardial and epicardial mapping and ablation), Sohns *et al*. [145] demonstrated acute procedural success rate of 90% and VT-free rate of 80% after a median follow-up of 9.5 months. In a recently published article including 42 patients (3 patients with combined endocardial and epicardial mapping and ablation), Sánchez Muñoz *et al*. [62] further classified these patients into isolated LVNC, LVNC with dilated cardiomyopathy, and LVNC with hypertrophic cardiomyopathy. Of note, they found that the arrhythmogenic substrates were heterogeneous, with origin in the ventricular outflow tracts and Purkinje system and scar patterns were similar to that in non-ischemic cardiomyopathy [62]. Furthermore, the VA-free rate at the end of the study was 40% [62].

## 5. Conclusions

In conclusion, the presence of VT has been observed in a variety of ACMs at an early stage, regardless of the severity of the disease [4]. Since VT circuits are commonly three-dimensional and epicardial substrates are frequently seen in ACMs, successful ablation may require both endocardial and epicardial approaches [10]. Pre-operative and intra-operative evaluation provides crucial information for identifying intramural or epicardial arrhythmogenic substrates and determining whether an epicardial approach is necessary [146]. Given the heterogeneous substrate characteristics, diverse disease progression, and various ablation strategies, outcomes are often variable in ACMs [4]. RFCA of ACM cannot be considered a substitute for ICD implantation based on current evidence. Therefore, further research is needed to better understand the mechanisms and ablation targets and to prevent disease progression.
